# New horizons at L’Anse aux Meadows

**DOI:** 10.1073/pnas.1907986116

**Published:** 2019-07-15

**Authors:** Paul M. Ledger, Linus Girdland-Flink, Véronique Forbes

**Affiliations:** ^a^Department of Archaeology, Queens College, Memorial University of Newfoundland, St. John’s, NL, Canada A1C 5S7;; ^b^Department of Geography, Memorial University of Newfoundland, St. John's, NL, Canada A1B 3X9;; ^c^School of Natural Sciences and Psychology, Liverpool John Moores University, L3 3AF Liverpool, United Kingdom

**Keywords:** Norse, indigenous, Bayesian modeling, insects, pollen

## Abstract

The UNESCO World Heritage site of L’Anse aux Meadows (LAM) in northern Newfoundland is the only undisputed site of pre-1492 presence of Europeans in the Americas. In August 2018, we undertook fieldwork at LAM to sample the peat bog 30 m east of the Norse ruins for a multiproxy paleoenvironmental assessment of Norse settlement. Instead, we encountered a new cultural horizon. Here we report our fieldwork at this iconic site and a Bayesian analysis of legacy radiocarbon data, which nuance previous conclusions and suggest Norse activity at LAM may have endured for a century. In light of these findings, we reflect on how the cultural horizon, containing nonnative ecofacts, may relate to indigenous or Norse activities.

The Norse colonization of the North Atlantic, a defining feature of the Viking Age, reached its ultimate extent on the shores of Newfoundland some 4 centuries before Christopher Columbus (1). Pre-Columbian presence of Europeans in the New World had often been suspected on the basis of oral histories inscribed by mid-13th century Icelandic scholars in *Grænlendinga Saga* and *Eiríks Saga Rauða*. These sagas recounted how Norse explorers from Greenland discovered and established a settlement in Vínland, a land west of Greenland (2). Nevertheless, it was not until the 1960s that definitive evidence was discovered near the Newfoundland outport of L’Anse aux Meadows (LAM) (1).

Throughout the 1960s, 8 Icelandic-style turf structures were excavated at LAM (Fig. 1), identifying Norse material culture such as a bronze cloak pin, a steatite spindle whorl, and evidence of rudimentary iron working (2). The remainder of the European material culture was nondiagnostic, comprising fragmentary iron, primarily nails and rivets, a bone needle fragment, and a whetstone (1). Numerous stone tools pertaining to indigenous peoples were also recovered (2), perhaps suggesting LAM was the theater of the earliest contact between Europeans and North Americans. In the 1970s, further excavations by Parks Canada revealed iron-worked wooden objects and jasper fire starters matching rocks that outcrop in western Greenland and Iceland (2). A wealth of wooden debitage was also uncovered, which was attributed to both indigenous and Norse occupations (2). Compared with Norse sites in Greenland, the limited suite of material culture from LAM is notable for the near absence of steatite and osseous material commonly used for all manner of implements in Greenland (Fig. 1). Botanical analyses identified wood and nuts from the White Walnut (*Juglans cinerea*), an exotic species in Newfoundland that suggests wider-ranging Norse voyages to the south (2).

**Fig. 1.**
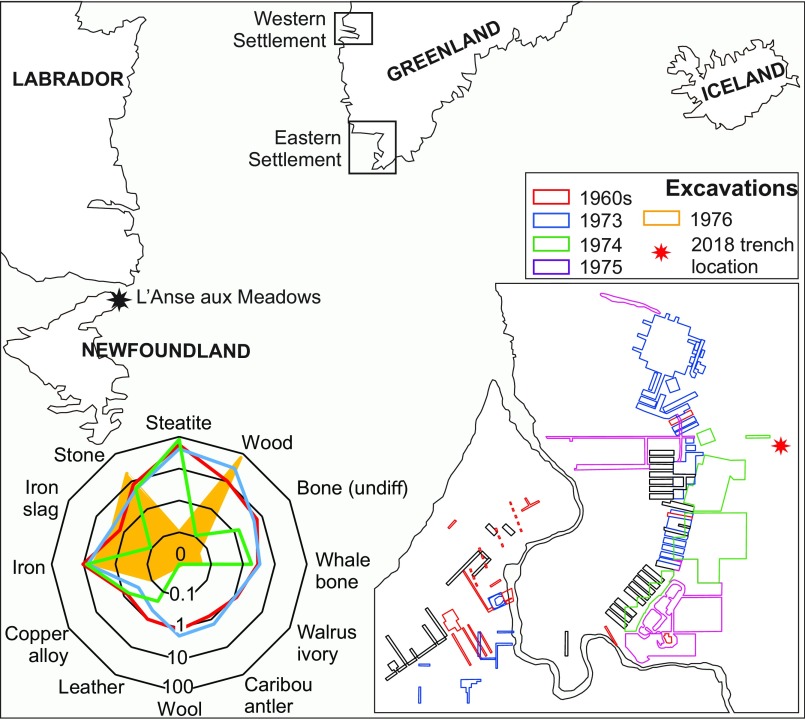
The North Atlantic region. (*Left Inset*) Summary of material culture from church farms (blue), farms (red), and shielings (green) in Greenland in terms of raw materials. The material culture assemblage from LAM is represented in yellow (*Right Inset*). The location of Norse ruins and excavations at LAM.

Over 100 ecofacts associated with the archaeological heritage (wood and charcoal) were submitted for radiocarbon dating (2). Dates ranged from 5,080 ± 110 ^14^C y B.P. (Qu-365; charcoal) to 260 ± 110 ^14^C y B.P. (S-1167; worked wood), encompassing the suite of occupations at the site. Assays from Norse contexts (56 assays) ranged from 1,630 ± 70 ^14^C y B.P. (WAT-420; charred wood) to 865 ± 65 ^14^C y B.P. (S-1091; wood). Icelandic saga literature, and references to Vínland in an ecclesiastical treatise from *ca*. AD 1075, suggested LAM should date to *ca*. AD 1000, something the radiocarbon data seemed to challenge (2). It was not until the “in-built” ages of materials such as wood and charcoal became widely recognized that these conflicting narratives could be reconciled. Assays on short-lived macrofossils (twigs) from Norse contexts dated to between 1,050 ± 65 ^14^C y B.P. (S-1340; *Abies* sp. twig; Cal AD 895–1030) and 955 ± 100 ^14^C y B.P. (S-1355; *Larix* sp. twig; Cal AD 995–1185), suggesting an occupation centered on AD 1000 (2). A weighted mean of twig dates—notwithstanding issues associated with combination of ^14^C ages from multiple individuals—provided a result of AD 986–1022 (3).

Despite being the earliest known European outpost in North America, LAM remains enigmatic. None of the structures are identifiable as animal shelters, nor is there faunal evidence for animal husbandry—the foundation of Norse subsistence in Greenland and Iceland (2). LAM is therefore an outlier within the Norse settlements of the North Atlantic. The paucity of material culture and shallow deposits indicate a transitory place functioning as a base for exploration of North America (2). Although the Norse colonization of the North Atlantic is often viewed as a search for farmland, it was also an endeavor to secure luxury resources for European markets (4). From this perspective, perhaps a Norse outpost makes perfect sense. LAM is located on the shores of a rich cod fishery—which, 6 centuries later, was home to hundreds of seasonal French fishers (5)—and in a dense nesting region for eiders (6). Both stockfish (dried cod) and eiderdown were highly prized commodities in the Viking Age (4, 7).

## Fieldwork

**Fig. 2.**
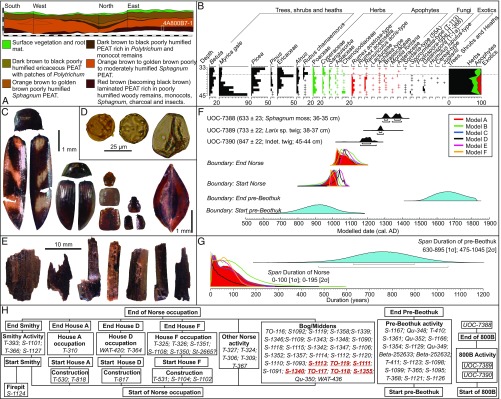
Results of analyses. (*A*) Trench stratigraphy and monolith sampling location. (*B*) Percentage pollen diagram displaying selected taxa (sum = ≥500 TLP), “+” indicates <1% TLP. (*C*) Insects and seeds from 4A800B7 (left to right): *Eanus macullipennis*, *S. metallica*, *A. quadrata*, *Pycnoglypta* sp.*,* and dock seed (cf. *R. aquaticus*). (*D*) Pollen (left to right): *H. lupulus*-type, *Juglans*, and cereal-type. (*E*) Wood debitage from 4A800B7. (*F*) Probability distributions for start and end of Norse and indigenous occupations and calibrated C^14^ assays from 4A800B7. (*H*) Modeled durations for Norse (red) and indigenous (blue) activity. (*G*) Prior information incorporated into all Bayesian models (*A*–*F*). Dates in italics were treated as outliers using the OxCal charcoal outlier model. Assays in bold red are short-lived elements also treated as outliers in models B, D, and F. *Terminus post quem* of 890 ± 60 in models C and D and 985 ± 1 in models E and F was included to indicate that Norse settlement at LAM occurred after the settlement of Iceland and Greenland, respectively.

Our trench, measuring 0.65 × 1.50 m, was located 30 m east of Ruin D (Fig. 1). The new cultural horizon (4A800B7) was encountered between 35 and 45 cm (Fig. 2) and comprised finely laminated (0.5 to 1.0 cm thick) apparently trampled surfaces containing charcoal, wood debitage, and charred plant remains. Abundant well-preserved plant (leaves and twigs) and insect remains were evident to the naked eye. A 2-L bulk sample of the layer and a monolith spanning the cultural horizon and natural peat were collected (Fig. 2).

## Environmental Archaeological Analyses

The peat monolith was cut into contiguous 1 cm^3^ subsamples, and the pollen content was analyzed (e.g., ref. 8). A 450-mL subsample of the 2-L bulk was disaggregated in <2% NaOH, then washed through a series of sieves. Residues were hand-sorted under a binocular microscope to isolate charcoal, seeds, insects, and wood debitage. Short-lived plant macrofossils selected from 3 levels (as illustrated in Fig. 2) were submitted for C^14^ dating at the André E. Lalonde Accelerator Mass Spectrometry Laboratory.

The pollen content is notable for tree, shrub, and heath percentages (in particular, *Myrica*), which are high in respect to the aspect of the site and previous studies at LAM (1, 9). Apophytes (e.g., *Rumex* sp. *Achillea millefolium* type) are elevated at *ca*. 10% total land pollen (TLP), while exotics such as *Juglans* (Walnut) and *Humulus* type (hops or cannabis) are also present. *Sporormiella*-type fungi, exclusively associated with the dung of grazing herbivores (caribou in the case of Newfoundland), were also found in 7 of 12 samples.

The concentration of insect remains in 4A800B7 (0.24 beetles per mL^−3^) is very high compared with natural peatlands. Most abundant are rove beetles such as *Pycnoglypta* sp., typically dominant on archaeological sites. Other examples include *Acidota quadrata* (Zetterstedt), a Holarctic species previously unrecorded in Newfoundland, and *Simplocaria metallica* (Sturm), a pill beetle considered adventive (nonnative) in Canada (10). Of further note is a dock/sorrel seed that compares favorably to *Rumex aquaticus* L., a species of uncertain status in Newfoundland (11).

## Bayesian Modeling of Radiocarbon Data

Legacy ^14^C data pertaining to the Norse and indigenous (often referred to as Recent Indian, a cultural group ancestral to the Beothuk) occupations at LAM were collated from the Canadian Archaeological Radiocarbon Database and placed within a relative chronological framework (Fig. 2) using published data (1, 2). A series of Bayesian models were constructed in OxCal (Fig. 2*F*) to estimate start and end dates for the Norse and indigenous occupations, individual structures, and 4A800B7 under varying prior information. Modeling results were remarkably consistent (Fig. 2*F*), and model A suggests Norse occupation began Cal AD 910–1030 (2*σ*; boundary: Start Norse), ended Cal AD 1030–1145 (2*σ*; boundary: End Norse), and endured for 0 to 195 y (2*σ*; span: Sequence Norse). Greater uncertainty surrounds indigenous occupations, where a start of Cal AD 710–1130 (2*σ*; boundary: Start pre-Beothuk) and end of Cal AD 1540–1815 (2*σ*; boundary: End pre-Beothuk) is indicated. Assays from 4A800B7 date it to the mid-12th to the late 13th centuries (Fig. 2).

## Discussion

Bayesian modeling has confirmed previous conclusions (2, 3) regarding Norse arrival, but suggests a potentially longer than assumed period of use (up to 195 y). This does not imply a continuous occupation, which, given the shallow cultural deposits, seems unlikely. Rather, it indicates the possibility of sporadic Norse activity beyond the early 11th century. Data from indigenous contexts is less precise, and activity is modeled to have begun between the 8th and 12th centuries. LAM therefore could have been a shared zone of interaction. Radiocarbon data place 4A800B7 in the 12th and 13th centuries, implying an association with indigenous activities.

Assuming an indigenous association poses the question: What process formed 4A800B7? The deposit is thick (≥10 cm), with excellent preservation and fine laminations, which indicate rapid accumulation and burial of trampled material. The concentrations of insects, wood debitage, charcoal, and weed pollen are suggestive of abundant organic waste. *Sporormiella*-type fungi also point to the incorporation of dung into the matrix of the deposit, or the nearby presence of grazing animals. Similar deposits are well documented from Greenlandic Norse sites (8), but contrast with shallow cultural layers from contemporary indigenous settings in Newfoundland.

The ecofactual content of 4A800B7 poses interesting biogeographical questions, but offers few clues to its cultural affinity. The dock seed (cf. *Rumex aquaticus*) may derive from an introduced plant native to Eurasia, but the tendency of *Rumex* species to hybridize complicates secure identification. Cereal-type pollen could derive from wild grasses, while *Humulus lupulus*-type and *Juglans* may have arrived through wind dispersal. However, their sole presence in the cultural horizon and the past identification of *Juglans* macrofossils at LAM is intriguing. Of the beetles, *S. metallica* is considered native to Greenland, where it has been found in Norse and Pre-Inuit contexts, while *A. quadrata*, never previously identified from Newfoundland, is common in the circumpolar north. If any of these species truly are introductions to Newfoundland, their arrival by the 13th century may have been via either Norse or indigenous trade or migration routes.

## Conclusions

The results presented here pose more questions than answers. The cultural deposit that we have discovered at LAM exhibits affinities with cultural layers from across the Norse North Atlantic, but our analyses demonstrate it postdates even the most generous estimates for Norse activity in Newfoundland. Ecofacts from cultural waste sandwiched between layers of peat may not be as evocative as artifacts such as a ringed bronze pin, or a finely crafted lithic projectile point. Yet, they present new horizons for examining the environmental legacies of inter- and intracontinental movement of people within North America prior to 1492. Whatever their affinity, the cultural deposits within the LAM peatlands preserve unique archaeological and biogeographical stories waiting to be told.
